# Ultra-Processed: The Search of Positioning From the Food Industry Regulatory Authorities

**DOI:** 10.3389/fnut.2022.906561

**Published:** 2022-06-06

**Authors:** Raul Amaral Rego

**Affiliations:** Certificates Programs, Insper Institute of Education and Research, São Paulo, Brazil

**Keywords:** processed food, nutritional content, additives, scientific research, public policies, regulatory authorities

## Introduction

There appears to be a global consensus on the importance of transforming the food system to provide current and future generations with nutritious and sustainable diets. The food industry and several of its stakeholders (1, 2) have similar interests in terms of guidelines and actions (3), such as improving nutritional value and reducing sugar, fat, and sodium content, reducing losses and waste and greenhouse gas emissions, and improving the efficiency of water usage in supply chains, among others.

However, other important stakeholders include the Food and Agriculture Organization of the United Nations (FAO), International Fund for Agricultural Development, United Nations Children's Fund, World Food Programme, World Health Organization, and philanthropic organizations from the Global Alliance for the Future of Food, they have been considering the need of deploying public policies to inhibit several categories of processed foods from having negative impacts on human health (4), including advocating the restriction of marketing and sales of foods classified as ultra-processed because they are high in calories and have minimal nutritional value (5).

The idea of prohibiting the consumption of different categories of processed foods, as they are associated with non-communicable chronic diseases, was reinforced with scientific studies proposing a form of food classification, called NOVA, in which the category of ultra-processed foods emerged (6, 7). NOVA began to gain greater relevance from the moment it began to be used in the elaboration of food guidelines, legislation on nutritional labeling, fiscal policies and proposals to restrict its promotion and commercialization, always based on several hypotheses about the reasons why such products would not be healthy. Therefore, Popkin et al. (8) consider the necessity of countries having “unified and impactful policies to reduce ultra-processed food consumption and promote healthier eating”, highlighting examples of nations that have adopted fiscal policies such as sugar-based taxes (South Africa, United Kingdom) and sugar-sweetened beverage taxes (Mexico, South Africa), front-of-package warning labels (Argentina, Brazil, Chile, Colombia, Costa Rica, Israel, Peru, Uruguay), marketing bans (Chile), school food policies (Brazil, Chile) and media campaigns (Chile, Mexico, South Africa).

Recently, in the period preceding COP26, this movement brought together several institutions that launched a series of proposals for changes in the food system, including restrictions on ultra or highly processed foods considering that, in addition to being harmful to health, they would also be unsustainable (9–12). The evidence linking trade agreements and food environments is analized by Friel et al. (13), taking into consideration that liberal foreign trade policies may have a negative repercussion on the control of non-communicable diseases, including obesity.

However, researchers in the field of food science and technology (14–24) have highlighted the existence of several contradictions and inconsistencies between the NOVA classification and the concept of ultra-processed food, which evidence the inadequacy of the use of this approach in the formulation of public policies, configuring a problem to be addressed so that changes in the food system can occur in a consensual way among its participants. This issue requires the positioning of regulatory authorities, so that questions related to the adoption of the NOVA classification as a basis for public policies can be resolved. In this direction, this work seeks to contribute to the identification of existing divergences, from a scientific point of view.

### Pressure for Using the NOVA Classification for Political Interventions in the Food Industry

The concept of ultra-processed food emerged from published works, such as Monteiro et al. (6) and Mourabac et al. (7), among others, which proposed classifying industrialized products based on their suitability for consumption. Initially restricted to health professionals, the NOVA classification proposal sparked an intense debate along with the industry when it was used as the foundation of a public policy in Brazil, the Dietary Guidelines for the Brazilian population (25). Since then, there has been a series of efforts from NOVA supporters to prove that ultra-processed are harmful, spreading the idea that they are responsible for a wide range of illnesses that afflict societies.

The first published papers on NOVA (6), highlighting the Dietary Guidelines for the Brazilian population (25), and a publication from Pan American Health Organization (26), had already claimed that ultra-processed foods were responsible for several chronic non-communicable diseases, as well as environmental, traditional culinary damages among others but without identifying the cause to all of them. Since then, there has been a crescent trend (27) in the development of scientific studies seeking to prove this affirmation, which was successively relating several industrialized food categories to low-quality diets (28), obesity, excessive calorie consumption and weight gain (29–31), diabetes type 2 (32, 33), heart diseases (34–36), different types of cancer (37–42), digestive system diseases (43), higher mortality risk (44–48), neurodegenerative diseases (49), and lower immunological tolerance (50), among other problems to human health such as hypertension, metabolic syndrome, depression, asthma (51). In addition to these papers linking allegedly ultra-processed foods to various diseases and health issues, other researchers attempted to connect them to negative impacts on the sustainability of the food system (10, 11) for its allegedly high greenhouse gas emissions and larger water footprints (11), among others.

These papers have served as sufficient justification for the proposal of political interventions (9) to transform the food system, such as the use of NOVA by public agents in food guidelines, taxation of ultra-processed foods, and educational campaigns to encourage people to avoid such products. Nonetheless, this movement has been questioned by multiple researchers in the field of food science and technology, which policymakers should take into consideration. Since the use of NOVA before being applied as desired by its defensors, its gaps should be widely debated from a technical-scientific and regulatory standpoint.

Baker et al. (52) identified the global growth trend in the demand for ultra-processed foods, as a problem that requires more adequate public policies to control the possible negative impacts on the nutrition and health of populations. However, there are empirical data that show the need to better study the relationship between the demand of industrialized food groups, notably those classified as ultra-processed, and non-communicable diseases, such as obesity.

A research made in Brazil by its Ministry of Health (53) has shown conflicting data regarding the direct relation of the demand of food considered ultra-processed and obesity. The research has identified the individuals who had consumed five or more ultra-processed food groups the day before the interview, estimating that they represented 18.5% of the adult population. However, the stratum with the highest consumption by age was that of individuals between 18 and 24 years old (32.4%), with the lowest percentage of obesity (9.9%). The same occurred with people between 25 and 34 years old, with 23.8% of consumers of ultra-processed foods and a percentage of obesity (19.6%) lower than the average of 21.5%.

Another survey by the Brazilian Ministry of Health (54), on the evolution of obesity and soft drink consumption, showed that the percentage of obese adults grew from 11.8% in 2006 to 21.5% in 2020, while the percentage of adults who consume soft drinks five or more days a week decreased from 30.9% in 2006 to 15.2% in 2020. Study by Barclay and Brand-Miller (55) revealed the existence of a paradox between the relationship between sugar intake with overweight and obesity in the Australian population, as they have inversely related trends over three decades, suggesting that “efforts to reduce sugar intake may reduce consumption but may not reduce the prevalence of obesity”.

The relation between food and diseases is much more complex. Despite pieces of evidence, wich reveals the health hazards that derivates from the excessive consumption of sugar, saturated fats, and sodium, and also from the low ingestion of essential nutrients such as fibers, is difficult to establish that the higher consumption of a determinate group of processed foods is, effectively, the cause of specific diseases, to the point that believing that restricting their consumption represents an efficient solution to represents an effective solution against the prevalence of diseases. Among certain processed food categories there might exist products that should have their consumption lowered so that individuals could maintain a more balanced diet. On the other hand, this diminishing could, singly, not assure a better diet, on the hypothesis of people maintaining the consumption of other food not processed with high-calorie density, not practice exercises among other habits considered unhealthy.

### Questions From Food Science and Technology Fields

Several researchers in food science and technology have presented their findings, which show flaws in the NOVA classification and the inadequacy of the ultra-processed food concept.

The relationship between food and diseases is highly disputed. According to studies by Eichermiller et al. (56) and Vergeer et al. (57), there is no evidence that the nutritional value and how healthy some foods are related to levels of processing because processed foods contribute to a wide range of nutrients in all levels of processing. According to Petrus et al. (20), the healthiness of a portion of food has nothing to do with the number of ingredients it contains or the intensity or quantity of processes used in its preparation, both of which are factors considered by Sadler et al. (58). However, it is not the number of processes that NOVA considers when classifying foods but the number of specific ingredients, among which can be highlighted those that would allow the identification of ultra-processed foods, such as sugar, fat, saturated fat, trans fat, sodium, additives, and industrial raw materials.

A critical aspect of NOVA classified several categories of industrialized food as ultra-processed, generally because they all had the same harmful composition. Some papers have observed how this erroneous premise characterizes the generic and arbitrary feature of the NOVA classification (22, 23). This arbitrariness is well configurated in the criteria its authors have used to exemplify manners of identifying ultra-processed foods. For example, some plain industrialized yogurt is classified as minimally processed, while some yogurt with the addition of sweets is considered ultra-processed. If a consumer adds sugar to plain yogurt, it does not pose a health risk if used sparingly. Generally, bread is minimally processed when it is made only with flour, water, salt, and baking powder, without emulsifiers, whey, and other ingredients that would make them ultra-processed. Simple corn flakes would be minimally processed, becoming processed with the addition of sugar and ultra-processed if they contained food color or flavor (59).

Therefore, processed food can be included in a NOVA classification depending on whether or not adding one or more ingredients is condemned. This represents a serious problem to be solved because even though the NOVA authors will demonstrate that not all items in a category are ultra-processed, the categories as a whole are used in their intervention proposals against industrialized foods. Thus, both research on supposedly ultra-processed foods, food guidelines based on NOVA, and public policy recommendations generally refer to product categories, associating them with health problems.

According to Ivens (27), this generic approach has practical implications that contradict NOVA's purposes, which are to provide healthier diets to the population, since it makes it difficult to identify, specifically, which foods would be necessary for a healthy diet. Jones e Clemens (18) observed incongruities in several definitions of the NOVA classification about the nutritional value of processed foods. A contradictory element is that it does not consider the nutritional composition of products; thus, it condemns processed foods that are essential nutrients for the population (16, 17). Drewnowski, Gupta and Darmon (60), and a large number of studies, coordinated by Rego, Vialta, and Madi found about the composition of many food categories that are considered as ultra-processed by NOVA, such as sliced bread (61), yogurt (62), juices (63), cookies (64), ice-cream (65), pizza (66), hamburgers (67), and pastry and pasta (68). These studies showed that there are several products, within each category of foods considered ultra-processed, with small amounts of sugars, saturated fats and sodium, and also with relevant amounts of protein and fiber.

For example, when considering industrialized sliced bread as ultra-processed, generically, there are products that, on average, contain higher levels of proteins and fibers than those baked in bakeries, considered healthier by NOVA, are ignored. Conversely, a large variety of sliced bread contains fewer calories, saturated fat, and sodium when compared to bread made in bakeries. Furthermore, if a person decides to be guided by NOVA to consume bread, they might have an opposite result than expected. However, many of the allegedly ultra-processed foods are necessary for a balanced diet and do not contain excessive amounts of sugar, fat, and sodium, as dietary experts have already recognized (24). According to Tobias and Hall (69), food classified as ultra-processed can have highly positive aspects in terms of food safety; thus, this classification should not guide individuals' choices.

Besides nutritional aspects, NOVA introduces contradictions when classifying, generically, several categories of industrialized food for their alleged use of additives and industrial substances such as milk or soy proteins, gluten, maltodextrin, invert sugar, dextrose, and fructose because this varies significantly among different items included in each category, with the aggravating factor that such ingredients are authorized for use in current legislation. For example, the studies from Rego, Vialta, and Madi found in a sample of 70 bread (61) that 35 products did not contain emulsifiers, and 60 did not have dyes. In all other studies, significant variations were observed regarding the use of other additives such as thickeners, flavorings, flavor enhancers, and other substances condemned by NOVA.

Besides influencing population food choices, NOVA tends to introduce errors in research aimed at establishing associations between food and diseases by failing to consider the diversity of industrialized products. These epidemiological studies present methodological limitations, such as the use of food inquiries that were not explicitly projected for this purpose and food composition tables that do not contemplate the wide variety of processed food available in the market (15). Another question is regarding the definition of ultra-processed food not being accepted universally, which can induce an error in interpreting the results of research that adopt this classification (21). For this and other reasons, it is not considered appropriate to use the NOVA in public policies (58), particularly in preparing the Food-Based Dietary Guidelines (14). There is a need for further research to more accurately investigate the association between the consumption of ultra-processed foods and health since the existing evidence is not yet convincing (19), disregarding junk food.

Thus, there are numerous concerns about the inadequacy of NOVA classification. However, they are often ignored simply by questioning the credibility of researchers, attributed to the fact that some of these professionals have links to the food industry (70), without considering the data that reveal contradictions and inconsistencies of NOVA. In this way, the tendency is to increase the amount of research on both sides, intensifying the antagonism in the debate over the existence or not of ultra-processed food. This movement appears to lead all to an expensive and unproductive path, which could generate a vicious circle of scientific production.

What would be the best direction?

### Discussion

Food industries face challenges that will require effort and investment to become more sustainable and competitive. Silva et al. (71) observed that they need to reinvent themselves to be more aligned with their stakeholders, especially with consumers' new demands.

Concerning the nutritional aspects, the recommendation that people consume more nutritious food and limit their consumption of foods with high sugar, fat, and sodium contents is without merit because many companies have been reformulating their products following the health and well-being trend. In this direction, there are also formal agreements from business associations with the government, like Brazil and other countries, to reduce added sugar, sodium, and saturated fat and eliminate trans fats in industrialized food. This was going on before the NOVA classification was created.

Conversely, NOVA raises a serious issue by implying that the use of additives, among other industrial ingredients, may transform certain foods into ultra-processed and potentially hazardous to health foods. The affirmation that additives are not healthy is explicit on the Dietary Guidelines for the Brazilian population (25), for which “...while each additive used in these products must be tested and approved by health authorities, the long-term health effects and the cumulative effect of exposure to various additives are not always well known.” Recent research (72, 73) regarding the presence of additives in food continues to assume that the presence of additives cocktails can have adverse impacts on health.

In Brazil, the Ordinance No. 540—SVS/MS, of October 27, 1997, which establishes the fundamental principles governing the use of food additives, states that a type of additive must be prohibited when there is evidence or suspicion that it is unsafe for consumption, or that it might interfere with the nutritional value of the food if it serves to adulterate the product to induce the consumer to deception or confusion (74). Therefore, considering the current standards, food science is at an impasse. Is the body of studies and research supporting the efficacy and safety of additives solid and consistent, representing state of the art, or are NOVA's advocates right?

The solution for this matter, which has gained large proportions, appears to deserve some brief positioning from the regulatory agencies, following an example in Spain. According to the scientific committee of the Spanish Agency for Food Safety and Nutrition (75), allegedly ultra-processed foods should not be associated with consumer health status, and further research on the potential negative effects of these products is required. Similarly, other authorities such as Brazil's ANVISA, Brazilian Health Regulatory Agency from the Ministry of Health, the FDA, Food and Drug Administration from the United States, and the EFSA, European Food Safety Authority, from Europe should be followed. Furthermore, regulatory agencies could mediate existing conflicts between researchers who defend and those who oppose the NOVA classification. Efforts to reach a consensus, neutrally and peacefully, even if it represents a profound revision of the concepts of both parties, would only bring benefits to science, the food system, and society as a whole.

## Final Remarks

The integration from stakeholders is treated as a critical success factor for enabling and realizing the necessary changes in the food system in a way to achieve efficient process governance. In contrast, it is not a simple task to harmonize visions and interests that are quite distinct and antagonized, as portrayed in this paper. As a result, food system leaders must have full authority to name a mediator agent to this process, a role suggested to regulatory authorities. These authorities must take a proactive stance at this time. Their positions, which are always backed up by scientific evidence, are still held in high regard by those who develop public policies in the fields of nutrition, food, and health.

## Author Contributions

RR conceived and wrote the original manuscript and approved it for publication.

## Conflict of Interest

The author provides services in projects sponsored by organizations from the food sector.

## Publisher's Note

All claims expressed in this article are solely those of the authors and do not necessarily represent those of their affiliated organizations, or those of the publisher, the editors and the reviewers. Any product that may be evaluated in this article, or claim that may be made by its manufacturer, is not guaranteed or endorsed by the publisher.
